# Neutrophil Gelatinase-Associated Lipocalin (NGAL) predicts renal injury in acute decompensated cardiac failure: a prospective observational study

**DOI:** 10.1186/1471-2261-12-8

**Published:** 2012-02-17

**Authors:** Stephen Macdonald, Glenn Arendts, Yusuf Nagree, Xiao-Fang Xu

**Affiliations:** 1 Centre for Clinical Research in Emergency Medicine, Western Australian Institute for Medical Research, Level 5, MRF Building, Rear 50 Murray Street, Perth, WA6000, Australia; 2 Emergency Medicine, Royal Perth Hospital, GPO Box X2213, Perth, WA6000, Australia; 3 Emergency Medicine, University of Western Australia, 35 Stirling Highway, Crawley, WA6009, Australia; 4 Emergency Medicine, Fremantle Hospital, PO Box 480, Fremantle, WA6959, Australia; 5 Cardiology, Royal Perth Hospital, GPO Box X2213, Perth, WA 6000, Australia

## Abstract

**Background:**

Acute Decompensated Cardiac Failure (ADCF) is frequently associated with deterioration in renal function. Neutrophil gelatinase-associated lipocalin (NGAL) is an early marker of kidney injury. We aimed to determine if NGAL measured at admission predicts in-hospital acute kidney injury (AKI) in ADCF.

**Methods:**

A prospective observational study measured NGAL and B-natriuretic peptide (BNP) from patients with ADCF presenting to two tertiary hospitals. Patients received standard care and were followed up daily as inpatients. ADCF was defined by PRIDE score ≥ 6 and AKI by RIFLE criteria.

**Results:**

One hundred and two patients (median age 80, IQR 69-84 years, 52% male) were enrolled. AKI developed in 22 (25%) of 90 for whom outcome data was available. Seven patients died. NGAL was significantly elevated in those who developed AKI versus those who did not (median 130 ng/ml *vs *69 ng/ml, *p *= 0.002). NGAL was also higher in those who died (median 136 ng/ml *vs *68 ng/ml, *p *= 0.005). AKI was significantly associated with risk of death (5/22 (23%) *vs *1/68 (1.5%), *p *= 0.001), but not length of hospital stay. NGAL significantly correlated with admission eGFR but not BNP. For prediction of AKI, NGAL > 89 ng/ml had sensitivity of 68% and specificity of 70% with area under the receiver operator characteristic (ROC) curve of 0.71 (0.58-0.84). After adjustment for baseline renal function, the odds ratio (OR) for AKI was 3.73 (1.26-11.01) if admission NGAL > 89 ng/ml.

**Conclusions:**

Elevated NGAL at admission is associated with in-hospital AKI and mortality in patients with ADCF. However, it has only moderate diagnostic accuracy in this setting.

## Background

Cardiac failure is frequently complicated by renal impairment and this is associated with worse outcome [1-3]. Renal insufficiency, defined by estimated glomerular filtration rate (eGFR) < 60 ml/min/1.73 m^2 ^had an overall prevalence of 57% in an analysis of hospitalised patients with heart failure, and was an independent predictor of mortality [4]. In another study of patients admitted to hospital with Acute Decompensated Cardiac Failure (ADCF), 27% had subsequent deterioration of renal function which was associated with increased mortality and length of stay [5].

Neutrophil gelatinase-associated lipocalin (NGAL) has been shown to be an early marker of acute kidney injury (AKI) in a number of settings [6-9]. Mechanisms of kidney injury in ADCF are multi-factorial, and in some instances may be exacerbated by therapy such as diuretics [10-12]. Identification of patients at risk of subsequent deterioration in renal function may allow for individualised therapy to mitigate this, for example by careful titration of loop diuretic doses and avoidance of potential nephrotoxins such as intravenous radiographic contrast media.

The purpose of this investigation was to determine whether measurement of NGAL at presentation to the Emergency Department (ED) could predict in hospital AKI in patients with ADCF, and to assess the utility of this marker in risk-stratification.

## Methods

### Design and setting

A prospective, observational, un-blinded study conducted in the Emergency Department (ED) of two University hospitals in Western Australia between January 2010 and January 2011.

### Population and patient selection

Patients presenting to the ED with symptoms consistent with ADCF during rostered research nurse hours (7 days per week 0700-2200) were screened for inclusion and underwent testing for BNP to enable calculation of PRIDE acute heart failure score [13]. A modified PRIDE score ≥ 6 (using a BNP cut-off of 400 pg/ml rather than NT-proBNP) was considered diagnostic for ADCF. The hospital discharge summary was reviewed to confirm diagnosis. Exclusion criteria were age < 18 years and end-stage renal failure on dialysis. Informed written consent was obtained from all patients; in patients whose clinical condition at presentation precluded informed discussion, we had provision for 'deferred consent' to enable processing of early blood samples. Ongoing participation in this circumstance required the agreement of the next-of-kin, with consent obtained from the patient as soon as their clinical condition allowed. The study complied with the Declaration of Helsinki and was approved by the Royal Perth Hospital Human Research Ethics Committee (Reference EC 2009/097).

### Biomarker analysis

Blood samples were taken when initial intravenous access was obtained and prior to any intravenous therapy. BNP and NGAL assays were undertaken immediately by trained research personnel, with the Biosite *Triage *device (Alere, Inverness Medical, Australia Inc) using whole blood. We did not measure urine NGAL as this particular assay is designed only for blood. These results were not made available to clinical staff. Additionally all patients had electrolytes, urea, creatinine and troponin tested on the Abbott Architect (troponin I) or Roche Elecsys (troponin T) laboratory analyser. The NGAL assay used had a lower limit of detection of 60 ng/ml, and a manufacturer quoted 95^th ^percentile of 149 ng/ml, which was used as a dichotomous cut off where appropriate for the purposes of categorical analysis. Baseline estimated glomerular filtration rate (eGFR) was calculated using the admission creatinine level by the Modification of Diet in Renal Disease (MDRD) formula [14].

### Inpatient follow up

Patients were followed up daily by a research nurse. Clinical data including vital signs, weight, fluid balance, and significant investigations or interventions were noted. Episodes of shock, sepsis, myocardial infarction (determined from hospital discharge summary and according to AHA/ESC criteria [15]), administration of intravenous contrast, inotropic or ventilatory support and renal dialysis were recorded. All medications administered and dose changes were recorded. An increase of 50% or more of baseline loop diuretic dose for at least 24 hours, or the introduction of one of these agents was considered clinically relevant. Potential nephrotoxic agents such as ACE inhibitors were also noted. Renal function was monitored by urine output and daily serum creatinine. Patients were followed for up to 7 days, or until death/hospital discharge, for determination of AKI. Mortality and duration of hospital stay data was obtained from clinical records.

### Outcome measures and sample size calculation

The primary study outcome was AKI developing during the inpatient stay, defined by RIFLE criteria [16], using the admission creatinine as baseline. RIFLE R (Risk) is defined as fall in GFR ≥ 25% or creatinine rise ≥ 50% from baseline, or a fall in urine output to < 0.5 ml/kg/hour for at least 6 hours. Secondary outcome measures were duration of hospital stay and in-hospital mortality. Sample size was determined assuming AKI would develop in 20% of patients and that elevation of NGAL > 149 ng/ml (95th percentile) would confer a relative risk of 4 times for this outcome. A total sample of 85 would be sufficient to demonstrate this with power 0.8 and α 0.05. We aimed to recruit 100 patients to allow for 15% margin for missing data etc.

### Statistical analysis

Continuous data is reported as mean ± SD or median + IQR for parametric and non- parametric distributions respectively and differences analysed by Student's t-test or Mann-Whitney U test as appropriate. Relationships between continuous variables were assessed by Spearman's rank correlation coefficient. For proportions, univariate analyses used Chi squared or Fisher's exact test. Receiver operator characteristic curves were plotted for NGAL, eGFR and creatinine at admission. To adjust for admission eGFR, we used logistic regression analysis. Goodness-of-fit was assessed using the Hosmer and Lemeshow test. All analyses were conducted using SPSS version 17 (SPSS Inc, Chicago, IL, USA). 95% confidence intervals are quoted and a p value < 0.05 considered significant.

## Results

**Figure 1:**
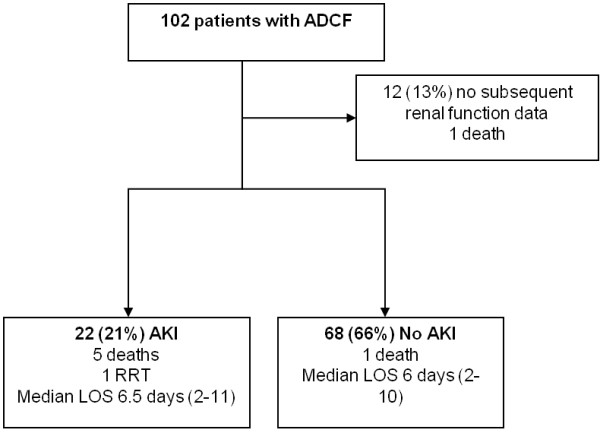
**Study participant flowchart. RRT - Renal Replacement Therapy, LOS - Length of Stay**.

One hundred and two patients were recruited into the study. Median age was 80 years (IQR 69-84 years) and 52 (51%) were male. Baseline characteristics of the study population are shown in Table 1. Twelve patients did not have subsequent data to enable determination of AKI. These patients were either transferred to another hospital after enrolment in the study, or were admitted but did not have subsequent creatinine measurement prior to discharge or death. Of the remaining 90 patients, 22 (25%) had in-hospital AKI including one patient who required short-term dialysis in the intensive care unit in the context of cardiogenic shock complicating myocardial infarction. On examination of the hospital discharge diagnosis, ADCF was subsequently not considered to be the primary diagnosis in 4 patients. None of these developed AKI. The flow of participants through the study is summarized in Figure 1.

**Table 1:** Participant characteristics and past medical history

n	102
Median Age (IQR)	80 (69-84)

Male	52 (51%)

Median BNP	723 pg/ml (IQR 337-1235 pg/ml)

Median PRIDE Score	12 (Range 6-14)

Congestive Cardiac Failure	50 (49%)

Hypertension	69 (68%)

Diabetes	46 (45%)

Coronary Heart Disease	60 (59%)

Valvular Heart Disease	19 (19%)

Chronic Obstructive Airways Disease	22 (22%)

Chronic Renal Impairment (admission eGFR < 60 ml/min/1.73 m^2^)	62 (60%)

### Relationship between NGAL, eGFR and BNP

There was a significant relationship between NGAL and admission eGFR (Spearman's rho -0.42, *p *= 0.01) but not between NGAL and BNP level (Spearman's rho +0.13, *p *= 0.18).

### NGAL and AKI

**Figure 2:**
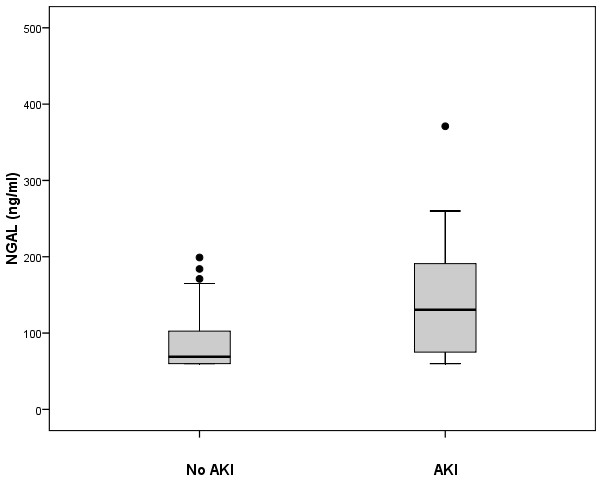
**Neutrophil gelatinase associated lipocalin (NGAL) and Acute Kidney Injury**. Median NGAL 130 ng/ml (IQR 71-193 ng/ml) in patients who subsequently developed AKI (n = 22) versus 69 ng/ml (IQR 60-103 ng/ml) for those who did not (n = 68), *p *= 0.002

Figure 2 compares admission NGAL level between those patients who developed in hospital AKI and those who did not. Median NGAL level was 130 ng/ml (IQR 71-193 ng/ml) for those who developed AKI compared to 69 ng/ml (IQR 60-103 ng/ml) for those who did not (*p *= 0.002).

### Mortality

Seven participants died during the hospital stay. Of these, five had AKI and one did not (*p *= 0.002). One further patient did not have subsequent renal function data to enable classification. Median NGAL was 136 ng/ml (IQR 133-170 ng/ml) for patients who died versus 68 ng/ml (IQR 60-119 ng/ml) for those who did not (*p *= 0.005).

### Length of hospital stay

Length of hospital stay was similar between those who developed AKI and those who did not. Median admission duration was 6.5 days (IQR 2-11 days) for the AKI group and 6 days (IQR 2-10 days) for those without AKI (*p *= 0.96). This did not significantly change when patients who died were excluded.

### Predictive value of NGAL for AKI

**Figure 3:**
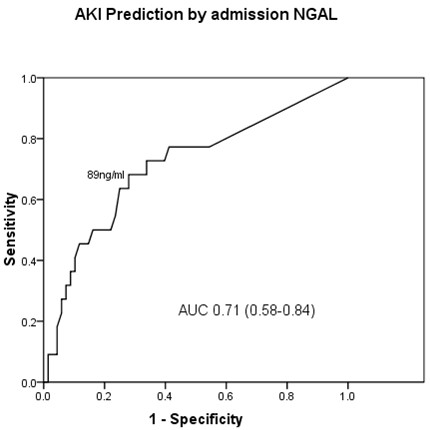
**Receiver operator characteristic (ROC) curve for prediction of AKI by NGAL measured at admission to hospital (AUC - area under the curve)**.

Table 2 shows results of univariate analysis of variables considered, *a priori*, likely to be associated with risk of developing AKI. The manufacturer quoted cut off at the 95th percentile for NGAL is 149 ng/ml. In the present study this value corresponded to a sensitivity of 45% and specificity of 85% for AKI. A receiver operator characteristic (ROC) curve (Figure 3) showed an optimal NGAL cut off at > 89 ng/ml, with sensitivity of 68% and specificity of 70% for AKI, with area under the curve (AUC) of 0.71 (0.58-0.84). The AUC of the ROC curve for admission eGFR was 0.72 (0.61-0.83) and for admission creatinine 0.70 (0.58-0.82). In addition to NGAL, baseline renal impairment (as defined by admission eGFR < 60 ml/min/1.73 m^2^) was significantly associated with AKI (Table 2). Four patients were subsequently considered not to have ADCF. All of these had NGAL < 89 ng/ml and none developed ADCF. Excluding these from the analysis did not significantly alter the results. After adjustment for admission eGFR, odds ratio (OR) for AKI was 3.73 (1.26-11.01) for NGAL > 89 ng/ml, and 4.78 (1.49-15.30) for NGAL > 149 ng/ml. NGAL was > 89 ng/ml in 14 of 19 patients (74%) with admission eGFR < 60 ml/min/1.73 m^2 ^who developed AKI, and in one of 3 (33%) of those with admission eGFR ≥ 60 ml/min/1.73 m^2 ^who developed AKI.

**Table 2:** Univariate analysis of risk factors for AKI

FACTOR	AKI	No AKI	Odds ratio (95%CI)	P value
NGAL > 89 ng/ml	15/22 (68%)	20/68 (29%)	5.1 (1.8-14.5)	0.003*

NGAL > 149 ng/ml	10/22 (45%)	8/68 (12%)	6.1 (2.0-18.8)	0.002*

Age ≥ 80 years	10/22 (45%)	33/68 (49%)	1.3 (0.4-3.3)	0.81

eGFR < 60 ml/min/1.73 m^2^	19/22 (86%)	35/68 (51%)	6.0 (1.6-22.0)	0.005*

Diabetes	10/22 (45%)	32/68 (47%)	0.9 (0.4-2.5)	0.90

Hypertension	16/22 (72%)	45/68 (66%)	1.4 (0.5-3.9)	0.57

Loop diuretic ↑^1^	15/22 (68%)	43/68 (63%)	1.2 (0.4-3.5)	0.64

Nephrotoxic drug^2^	7/22 (32%)	25/68 (37%)	0.8 (0.3-2.2)	0.64

IV Contrast	6/22 (27%)	15/68 (22%)	1.3 (0.4-4.0)	0.64

Sepsis	1/22 (4%)	1/68 (1.5%)	3.2 (0.2-54.0)	0.43

Shock	2/22 (9%)	4/68 (6%)	1.6 (0.3-9.4)	0.63

Myocardial Infarction	6/22 (27%)	9/68 (13%)	2.5 (0.8-8.0)	0.19

Urinary Infection	3/22 (14%)	5/68 (7%)	2.0 (0.4-9.1)	0.40

*p < 0.05

1 Introduction of loop diuretic or increase in usual dose of ≥ 50% over baseline for at least 24 hours (including bolus doses)

2 At least one dose of agent with potential renal toxicity including ACE inhibitor, angiotensin receptor blocker, non-steroidal anti-inflammatory drug, spironolactone, aminoglycoside antibiotic *etc*.

## Discussion

The present study confirms that AKI is a common complication of ADCF. Median NGAL is significantly higher at admission in patients who developed AKI and in those who died. Elevated NGAL at admission is associated with AKI after adjustment for eGFR. AKI is associated with increased risk of death but we did not find any association with increased length of hospital stay as has been found by other investigators [5]. This may have been a chance finding as the study was not powered to assess these outcomes. Although NGAL identifies a higher risk subgroup of patients for development of AKI, in this study it was found to have only moderate diagnostic accuracy.

Aghel *et al *[17] obtained NGAL levels on 91 admitted patients with physician adjudicated ADCF. Thirty eight percent of these developed worsening renal function (WRF), defined by a rise in creatinine > 0.3 mg/dl, with NGAL > 140 ng/ml having an odds ratio of 7.4 for WRF, after adjustment for baseline eGFR. Higher NGAL levels also increased risk of death. This study found admission NGAL to have a sensitivity of 85% and specificity of 54%, compared to 68% and 70% in our study, and found a higher NGAL cut off value. There were important differences from our study which may explain these discrepancies. We enrolled patients on arrival in the ED using a pragmatic diagnostic tool rather than selected by physicians after admission, when diagnostic workup may be more complete. The incidence of renal injury was lower in our study (25% *vs *38%) suggesting possible differences in illness severity, but there was a high prevalence of renal impairment at admission, possibly reflecting the higher median age in our study of 80 years. Finally the assays used were not the same. Differences in calibration of assays and antibodies used may result in markedly different NGAL cut off values.

Unlike other conditions such as post surgery or intravenous contrast administration where NGAL has be shown to be useful for predicting AKI, the time of onset of the renal insult is not easily determined in ADCF. Sixty percent of patients in our study had admission eGFR < 60 ml/min/1.73 m^2^. In the ED setting it is not clear whether this represents chronic impairment or acute injury unless information on stable baseline renal function is available. In some patients, low GFR at admission may represent AKI at its nadir, with subsequent recovery. Eight patients with NGAL > 149 ng/ml did not develop AKI as defined in this study. Some of these may have had renal injury when compared to their stable baseline eGFR rather than that recorded on admission. In addition, chronic renal impairment itself is a major risk factor for AKI and it is not surprising that low eGFR was significantly associated with this outcome. This finding alone, in the absence of elevated NGAL, should alert the clinician to a high risk of subsequent worsening renal function.

The mechanisms of renal injury in cardiac failure, also termed cardio-renal syndrome, are complex [10-12]. Renal hypoperfusion due to reduced cardiac output or increased venous or abdominal pressures may contribute. In addition high dose diuretics, intravenous contrast and other drugs may result in renal injury. These factors may explain why some patients who developed AKI in our study did not have elevated NGAL on presentation. NGAL has been shown to be elevated in patients with stable chronic heart failure [18]. While known to be released by renal tubular cells, expression of NGAL by cardiac myocytes has also been demonstrated in heart failure [19]. However a recent clinical study by Shrestha *et al *[20] found no association between NGAL level and echocardiographic parameters of systolic dysfunction.

### Limitations

Limitations of this study include its un-blinded and observational nature. Data to assess subsequent renal function was not available for all patients. This was most commonly due to no subsequent bloods being taken due to early discharge from hospital or transfer to another facility after recruitment in the ED. In addition, we were restricted in our ability to recruit overnight, a time when acute heart failure presentations are common. The diagnosis of ADCF was based on the clinical judgement of the treating clinician and the PRIDE score rather than formal echocardiography. We used BNP rather than NT-BNP to calculate this score. A BNP > 400 pg/ml cut point was chosen based on manufacturer recommendation however its incorporation into the PRIDE score has not been validated. This was a pragmatic study reflecting real life clinical practice where initial treatment of ADCF is undertaken in the ED. We did not have information on baseline renal function and used worsening from admission to define AKI with a single NGAL assay. However, ADCF is a dynamic condition and patients present to hospital at varying stages of the illness; obtaining serial NGAL levels, and knowledge of stable baseline renal function prior to acute decompensation would allow further exploration the timing of NGAL peak in relation to nadir of renal function.

### Potential clinical implications

Potential benefits of being able to identify patients at risk of worsening renal function in ADCF are the ability to modify treatment regimes to mitigate this risk. However, a single NGAL level at admission is only moderately accurate, although high levels are associated with higher risk of AKI. The incremental value of NGAL in addition to eGFR estimation in identifying high-risk patients is uncertain. A statistical association with an outcome does not necessarily translate into clinical utility for a biomarker when added to existing clinical and laboratory risk factors [21]. Further studies are required to determine whether knowledge of NGAL level at admission translates into improved outcomes. NGAL may be a tool to unravel the underlying pathophysiology of the cardio-renal syndrome.

## Conclusions

Elevated NGAL at admission is associated with in-hospital AKI, independent of baseline renal function, in patients admitted from the ED with ADCF and is associated with increased mortality. However, it has only moderate diagnostic accuracy in this setting.

## Competing interests

SPJM and YN have both spoken at meetings sponsored by Alere (Inverness Medical Innovations Australia Pty). No honoraria or travel costs were claimed. GA and X-FX declare no competing interests. All authors are employees of the Health Department of Western Australia and/or University of Western Australia.

## Authors' contributions

SM had the original study idea and designed the study with GA and X-FX. All authors undertook data collection and oversaw enrolments. SM and GA undertook the analysis. SM drafted the paper and all authors contributed to its revision. All authors read and approved the final manuscript.

## Authors' information

SM: Emergency Physician and Associate Professor; GA: Emergency Physician and Associate Professor; YN: Emergency Physician and Professor; X-FX: Cardiologist.

## Pre-publication history

The pre-publication history for this paper can be accessed here:

http://www.biomedcentral.com/1471-2261/12/8/prepub

## References

[B1] Adams KF Jr Fonarow GC Emerman CL LeJemtel TH Costanzo MR Abraham WT Berkowitz RL Galvao M Horton DP Characteristics and outcomes of patients hospitalized for heart failure in the United States: rationale, design, and preliminary observations from the first 100,000 cases in the Acute Decompensated Heart Failure National Registry (ADHERE) Am Heart J 2005 149 2 209 216 10.1016/j.ahj.2004.08.005 15846257

[B2] Hillege HL Nitsch D Pfeffer MA Swedberg K McMurray JJ Yusuf S Granger CB Michelson EL Ostergren J Cornel JH Renal function as a predictor of outcome in a broad spectrum of patients with heart failure Circulation 2006 113 5 671 678 10.1161/CIRCULATIONAHA.105.580506 16461840

[B3] Smith GL Lichtman JH Bracken MB Shlipak MG Phillips CO DiCapua P Krumholz HM Renal impairment and outcomes in heart failure: systematic review and meta-analysis J Am Coll Cardiol 2006 47 10 1987 1996 10.1016/j.jacc.2005.11.084 16697315

[B4] Amsalem Y Garty M Schwartz R Sandach A Behar S Caspi A Gottlieb S Ezra D Lewis BS Leor J Prevalence and significance of unrecognized renal insufficiency in patients with heart failure Eur Heart J 2008 29 8 1029 1036 10.1093/eurheartj/ehn102 18339607

[B5] Forman DE Butler J Wang Y Abraham WT O'Connor CM Gottlieb SS Loh E Massie BM Rich MW Stevenson LW Incidence, predictors at admission, and impact of worsening renal function among patients hospitalized with heart failure J Am Coll Cardiol 2004 43 1 61 67 10.1016/j.jacc.2003.07.031 14715185

[B6] Mishra J Dent C Tarabishi R Mitsnefes MM Ma Q Kelly C Ruff SM Zahedi K Shao M Bean J Neutrophil gelatinase-associated lipocalin (NGAL) as a biomarker for acute renal injury after cardiac surgery Lancet 2005 365 9466 1231 1238 10.1016/S0140-6736(05)74811-X 15811456

[B7] Constantin JM Futier E Perbet S Roszyk L Lautrette A Gillart T Guerin R Jabaudon M Souweine B Bazin JE Plasma neutrophil gelatinase-associated lipocalin is an early marker of acute kidney injury in adult critically ill patients: a prospective study J Crit Care 2010 25 1 176 e1-6 19781900 10.1016/j.jcrc.2009.05.010

[B8] Haase-Fielitz A Bellomo R Devarajan P Story D Matalanis G Dragun D Haase M Novel and conventional serum biomarkers predicting acute kidney injury in adult cardiac surgery-a prospective cohort study Critical care medicine 2009 37 2 553 560 10.1097/CCM.0b013e318195846e 19114878

[B9] Tuladhar SM Puntmann VO Soni M Punjabi PP Bogle RG Rapid detection of acute kidney injury by plasma and urinary neutrophil gelatinase-associated lipocalin after cardiopulmonary bypass J Cardiovasc Pharmacol 2009 53 3 261 266 10.1097/FJC.0b013e31819d6139 19247188

[B10] Sarraf M Masoumi A Schrier RW Cardiorenal syndrome in acute decompensated heart failure Clin J Am Soc Nephrol 2009 4 12 2013 2026 10.2215/CJN.03150509 19965544

[B11] Tang WH Mullens W Cardiorenal syndrome in decompensated heart failure Heart 2009 96 4 255 260 19401280 10.1136/hrt.2009.166256

[B12] Ronco C Haapio M House AA Anavekar N Bellomo R Cardiorenal syndrome J Am Coll Cardiol 2008 52 19 1527 1539 10.1016/j.jacc.2008.07.051 19007588

[B13] Baggish AL Siebert U Lainchbury JG Cameron R Anwaruddin S Chen A Krauser DG Tung R Brown DF Richards AM A validated clinical and biochemical score for the diagnosis of acute heart failure: the ProBNP Investigation of Dyspnea in the Emergency Department (PRIDE) acute heart failure score Am Heart J 2006 151 1 48 54 10.1016/j.ahj.2005.02.031 16368291

[B14] Levey AS Coresh J Greene T Stevens LA Zhang YL Hendriksen S Kusek JW Van Lente F Using standardized serum creatinine values in the modification of diet in renal disease study equation for estimating glomerular filtration rate Ann Intern Med 2006 145 4 247 254 16908915 10.7326/0003-4819-145-4-200608150-00004

[B15] Thygesen K Alpert JS White HD Universal definition of myocardial infarction Eur Heart J 2007 28 20 2525 2538 17951287 10.1093/eurheartj/ehm355

[B16] Bellomo R Ronco C Kellum JA Mehta RL Palevsky P Acute renal failure - definition, outcome measures, animal models, fluid therapy and information technology needs: the Second International Consensus Conference of the Acute Dialysis Quality Initiative (ADQI) Group Crit Care 2004 8 4 R204 212 10.1186/cc2872 15312219 PMC522841

[B17] Aghel A Shrestha K Mullens W Borowski A Tang WH Serum neutrophil gelatinase- associated lipocalin (NGAL) in predicting worsening renal function in acute decompensated heart failure J Card Fail 2010 16 1 49 54 10.1016/j.cardfail.2009.07.003 20123318 PMC2856952

[B18] Damman K van Veldhuisen DJ Navis G Voors AA Hillege HL Urinary neutrophil gelatinase associated lipocalin (NGAL), a marker of tubular damage, is increased in patients with chronic heart failure Eur J Heart Fail 2008 10 10 997 1000 10.1016/j.ejheart.2008.07.001 18804416

[B19] Yndestad A Landro L Ueland T Dahl CP Flo TH Vinge LE Espevik T Froland SS Husberg C Christensen G Increased systemic and myocardial expression of neutrophil gelatinase-associated lipocalin in clinical and experimental heart failure Eur Heart J 2009 30 10 1229 1236 10.1093/eurheartj/ehp088 19329498

[B20] Shrestha K Borowski AG Troughton RW Thomas JD Klein AL Tang WH Renal dysfunction is a stronger determinant of systemic neutrophil gelatinase-associated lipocalin levels than myocardial dysfunction in systolic heart failure J Card Fail 2011 17 6 472 478 10.1016/j.cardfail.2011.02.003 21624735 PMC3105247

[B21] Pencina MJ D'Agostino RB Sr D'Agostino RB Jr Vasan RS Evaluating the added predictive ability of a new marker: from area under the ROC curve to reclassification and beyond Statistics in medicine 2008 27 2 157 172 discussion 207-112 10.1002/sim.2929 17569110

